# An Index Rerum of Dental Literature

**Published:** 1878-12

**Authors:** D. C. Hawxhurst


### AN INDEX RERUM OF DENTAL LITERATURE.

BY D. C. HAWXHURST, D. D. S.

The man who does not read dental literature will probably care very little about an index of the same. The suggestion which I am about to make is designed for the benefit of those practitioners of dentistry who read more or less of the book and magazine literature of our profession, and who might wish, on any given occasion, to consult off hand and at a moment's notice, whatever writings are in existence relative to any particular topic. The literature of most topics is scattered through different books or chapters, and widely disseminated through the various magazines of the day.

The plan is one which involves the making of an entry in a book prepared for the purpose, every time an article has been read containing important suggestions on any topic whatever.

As the name, index rerum, indicates, it is simply an index of readings. The busy practitioner must have observed, that at the very time when he wishes to make use of some important fact or principle that he remembers reading some where, he often forgets where he read it. By means of an accurately kept index of his readings he would be able to turn at a moment's notice to every thing which he had read on the subject, or to any special article.

Suppose, for instance, that a patient presents with a tooth whose crown can not be saved with filling. It is desirable to mount a crown on the root. For some reasons the ordinary method of pivoting is contra-indicated, and can not be employed. The practitioner wishes now to refresh his memory on rare and unusual methods of pivoting. Descriptions of these are to be found throughout the various volumes of the DENTAL RLGISTER, Dental Cosmos, Dental Miscellany, Dental News-letter, and in every other dental journal, ancient or modern, in this country or in Europe, and probably in a number of books also. If the practitioner is a very old one he will have read the most of these, will have indexed them under the head of "Pivoting Teeth," and will in the main have forgotten them. But, on reading their titles, he will recall enough of each, especially the more modern ones to enable him to determine which to re-read. Often he will go to his index rerum, remembering exactly what he wants, and only seeking the name and number of the journal which contains it. Or he may desire to make an exhaustive study of the whole subject, and will then go to his index as a guide to its literature. By means of such an index, every important subject, from neuralgia to vitiated oral secretions, from alveolar abscess to dentigerous cysts may be at once referred to and everything that has been read on the subject brought under view.

In this case each practitioner makes his own index rerum; he knows what his own purposes are, and will not index an article unless it contain something that he believes will be of use to him in the future. He may leave out .two-thirds of the articles written on any topic, either because they are in themselves worthless, or because whatever is good in them is embodied in other and abler articles already indexed.

This will prove a great saving of labor, when in future years he desires to investigate any subject thoroughly and with the least possible expenditure of time and effort. He may still further economize time by indicating in his index the important paragraphs to be consulted.

Such an index can not but be a valuable aid, not only to the writer and investigator, but to the practitioner in his office and laboratory. Indeed, no professional man can afford to be without it, for it gives him a wide and ready command of all that he has read. It does not propose to preserve for him important notes and hints drawn from his reading, which indeed would be a highly useful result; it does more; it enables him to refer at once to the original article and point out the important paragraphs. It does this by noting for his use qıəcisely what no living man can himself remember, the name, year of issue, number and page of the periodical or dook containing what he seeks.

In my opinion every dentist who is about to begin the practice of his profession and has procured the ordinary stock of instruments for that purpose, should make three additions to this outfit—a wife, a microscope and an index rerum.

Passing over the first two, I should say that he will find the index rerum of John Todd highly suitable for his purpose. It is a well bound volume of several hundred blank leaves, lettered for the convenient entry of subjects, and containing a preface which fully explains how to use it. It may be ordered from any bookseller, at a cost of two or three dollars. An ordinary blank book may be made to answer a similar purpose. Even a small blank book that shall cost but the trifling sum of twenty-five cents can be made to serve an excellent purpose if the reader is really a poor man. Or a quire of paper may be purchased for a dime and stiched at the back, which will give forty-eight pages. At the top of each page may be written some important subject. Here is an index rerum which will last till the young practitioner has grown older and richer.

